# Clinical Diagnostic Performance of Droplet Digital PCR for Suspected Bloodstream Infections

**DOI:** 10.1128/spectrum.01378-22

**Published:** 2023-01-05

**Authors:** Ke Lin, Yuanhan Zhao, Bin Xu, Shenglei Yu, Zhangfan Fu, Yi Zhang, Hongyu Wang, Jieyu Song, Mingxiang Fan, Yang Zhou, Jingwen Ai, Chao Qiu, Haocheng Zhang, Wenhong Zhang

**Affiliations:** a Department of Infectious Disease of Huashan Hospital, National Medical Center for Infectious Diseases and Shanghai Key Laboratory of Infectious Diseases and Biosafety Emergency Response, Fudan University, Shanghai, China; b Key Laboratory of Medical Molecular Virology (MOE/MOH) and Institutes of Biomedical Sciences, Shanghai Medical College, Fudan University, Shanghai, China; c State Key Laboratory of Genetic Engineering, School of Life Science, Fudan University, Shanghai, China; d National Clinical Research Centre for Aging and Medicine, Huashan Hospital, Fudan University, Shanghai, China; University of Pittsburgh

**Keywords:** antibiotic application, bloodstream infection, clinical application, digital droplet PCR, etiological diagnosis, medicine regimens

## Abstract

Accurate and timely etiological diagnosis is crucial for bloodstream infections (BSIs) due to their high disability and mortality. We conducted a single-center prospective cohort study to compare the digital droplet PCR (ddPCR) assay with traditional blood culture. A total of 169 blood samples from 122 patients with suspected BSIs were collected, mostly from the department of infectious diseases, the emergency department, and the intensive care units, and the clinical data were also recorded. Nucleic acid was extracted from the blood samples, and a 5-fluorescent-channel droplet digital PCR assay was performed and then fed back with the pathogen and its copies. In BSI patients, ddPCR reported an overall 85.71% (12/14) (95% confidence interval [CI], 56.15 to 97.48%) sensitivity, 100% (7/7) (95% CI, 56.09 to 100.00%) and 71.43% (5/7) (95% CI, 30.26 to 94.89%) sensitivity in patients without empirical treatment and in empirically treated patients, respectively. Compared to traditional blood culture, the overall detection rate of ddPCR was significantly higher, 11.27% (16/142) (95% CI, 6.78 to 17.93%) versus 30.28% (43/142) (95% CI, 23.01 to 38.64%), and the extra detection rate of ddPCR was 19.01% (27/142) (95% CI, 13.11 to 26.63%). Of the ddPCR-positive culture-negative cases, 74.19% (23/31) (95% CI, 55.07 to 87.46%) were consistent with the final clinical diagnosis, including 10 bacteria and fungi. The detection rate of ddPCR was significantly higher in patients with white blood cell (WBC) counts of >10 · 10^9^/L, C-reactive protein (CRP) of >70 mg/L, or procalcitonin (PCT) of >0.9 ng/L. Pathogen loads detected by ddPCR are correlated with WBC, CRP, and especially, PCT levels, precisely and rapidly reflecting clinical disease progression. ddPCR has an important guiding value for the clinical use of antibiotics to achieve the best pathogen coverage and the antibacterial effect. Collectively, ddPCR showed a great diagnostic performance in BSIs and had an overall higher detection rate than blood culture. In addition, ddPCR could be used to dynamically monitor the disease progression and provide medication guidance on antibiotic use.

**IMPORTANCE** ddPCR is a promising method to address the current challenges of BSI diagnosis and precise treatment, as it is highly efficient in DNA detection. It shortens the identification of BSI-related pathogens from several days of traditional bacterial culture to 4 to 5 h. It is extremely sensitive and more tolerant to PCR inhibitors, which may facilitate the amplification and enable the detection of a meager amount of DNA fragments in detecting BSI-related pathogens and drug-resistant genes. It can identify almost 20 pathogens in one reaction, which reduces the usage of clinical blood samples to no more than 2 mL. Additionally, dynamic monitoring, assessment of pathogens, and antibiotic resistance genes in patients could be used to guide timely and precise adjustment of antimicrobial prescription. The short turnaround time of ddPCR may have the potential to guide antimicrobial treatment in the very early stage of sepsis and reduce the mortality and disability rate of sepsis.

## INTRODUCTION

Bloodstream infections (BSIs) are a significant public health concern worldwide, with high morbidity and mortality, particularly those causing sepsis (1, 2). Delayed initiation of effective antimicrobial therapy leads to worsened clinical outcomes, unnecessary broad-spectrum antibiotic usage, and increasing health care costs. In contrast, fast and accurate identification of causative pathogens with antimicrobial susceptibility testing (AST) could lead to a more precise therapy, improving clinical outcomes (3, 4).

The diagnosis of BSIs strongly relies on the amount of pathogen in the blood. Currently, blood culture with AST is the gold standard and first-line tool for diagnosing BSIs. However, blood culture is limited by a long turnaround time (TAT), taking around 6 h to 5 days to grow an organism to detectable levels, with additional time required to identify the organism and provide AST results. In most cases, a delay in pathogen identification prevents a timely switch from broad-spectrum antibiotics to targeted therapy. According to previous research, during the empirical treatment phase before the microbiological culture results are available, over 70% of BSI patients with fungemia receive inappropriate therapy. Inadequate long-term use of broad-spectrum antibiotics can result in drug toxicity, antimicrobial drug resistance, an increase in hospital readmissions, and higher expenses for both patients and the health care system (5). It is also relatively insensitive after initiation of antibiotic therapy and can sometimes be misleading because of contamination (6, 7).

Until now, several molecular rapid diagnostic tests (mRDTs) have been developed, and each emerging technology has its unique benefits and drawbacks. Among these methods, the universal goal is to ensure satisfactory sensitivity detection and shorten the diagnostic timeline. Molecular diagnostic tests directly applied to whole blood without culture, such as next-generation sequencing, have been shown to significantly decrease the TAT compared to conventional methods, resulting in the more rapid implementation of pathogen-directed antimicrobial therapy, shortened length of hospital stay, and possibly, reduced mortality in the presence of antimicrobial stewardship programs (8). Most molecular amplification methods based on real-time quantitative PCR, microarray technology, and nanoparticle-based assays such as SeptiFast (9) shorten the turnaround time to 6 to 10 h with less hands-on time. Metagenomic next-generation sequencing (mNGS) achieves a wide breadth of detection. However, this method is often not sensitive enough to detect a low amount of DNA fragments of BSI-related pathogens and drug-resistant genes (10).

Droplet digital PCR (ddPCR) is a novel one-step PCR assay and has been proven to achieve higher accuracy and sensitivity than qPCR. It also stands out as a method of absolute nucleic acid quantification by using Poisson statistical analysis of the number of positive and negative droplets. Therefore, external references with variations in PCR efficiency are not necessary anymore (11). It has been used in a lot of clinical fields, such as liquid biopsies for cancer monitoring and noninvasive prenatal testing for genetic abnormality detection (12–14). Recently, ddPCR has shown promising potential in resolving polymicrobial infection since it simultaneously achieves unprecedented high sensitivity (able to detect pathogens at low concentrations of as few as 10 CFU/mL) in an about 4-h turnaround time (15).

There have been several studies evaluating the diagnostic accuracy of ddPCR. Wouters et al. (16) applied ddPCR to 45 blood samples collected from patients suspected of BSI, and the overall sensitivity and specificity of ddPCR compared with blood culture was 80% (95% confidence interval [CI], 52 to 96%) and 87% (95% CI, 69 to 96%) respectively. Zhou et al. (17) evaluated the ddPCR assay for pleural and peritoneal fluid samples and the sensitivity was 96% (95% CI, 79.65 to 99.90%) and 92.86% (95% CI, 66.13 to 99.82%), respectively. Furthermore, Hu et al. (18) performed a head-to-head comparison between ddPCR and mNGS in 45 critically ill patients. They found that within the target pathogen range of the ddPCR assay, it showed a higher detection rate of blood pathogens than the mNGS assay (88 positives in ddPCR versus 53 positives in mNGS), while the range of pathogens detected by plasma DNA mNGS (*n* = 126) was wider than that detected by ddPCR (*n* = 88). However, the diagnostic accuracy of suspected BSIs as well as the clinical impact of the ddPCR-based method on therapeutic decision has not yet been well studied (15, 16).

This research evaluated the diagnostic accuracy of the ddPCR and examined its consistency with conventional blood culture in a prospective cohort of suspected BSIs. We focused on 43 ddPCR-positive cases and analyzed the association between quantitative pathogen loads detected by ddPCR and laboratory results, including C-reactive protein (CRP), white blood cells (WBC), and procalcitonin (PCT), exploring the potential of ddPCR in monitoring clinical disease progression. Moreover, we further analyzed the application value of ddPCR in providing antimicrobial guidance in BSIs.

## RESULTS

### General characteristics.

**FIG 1:**
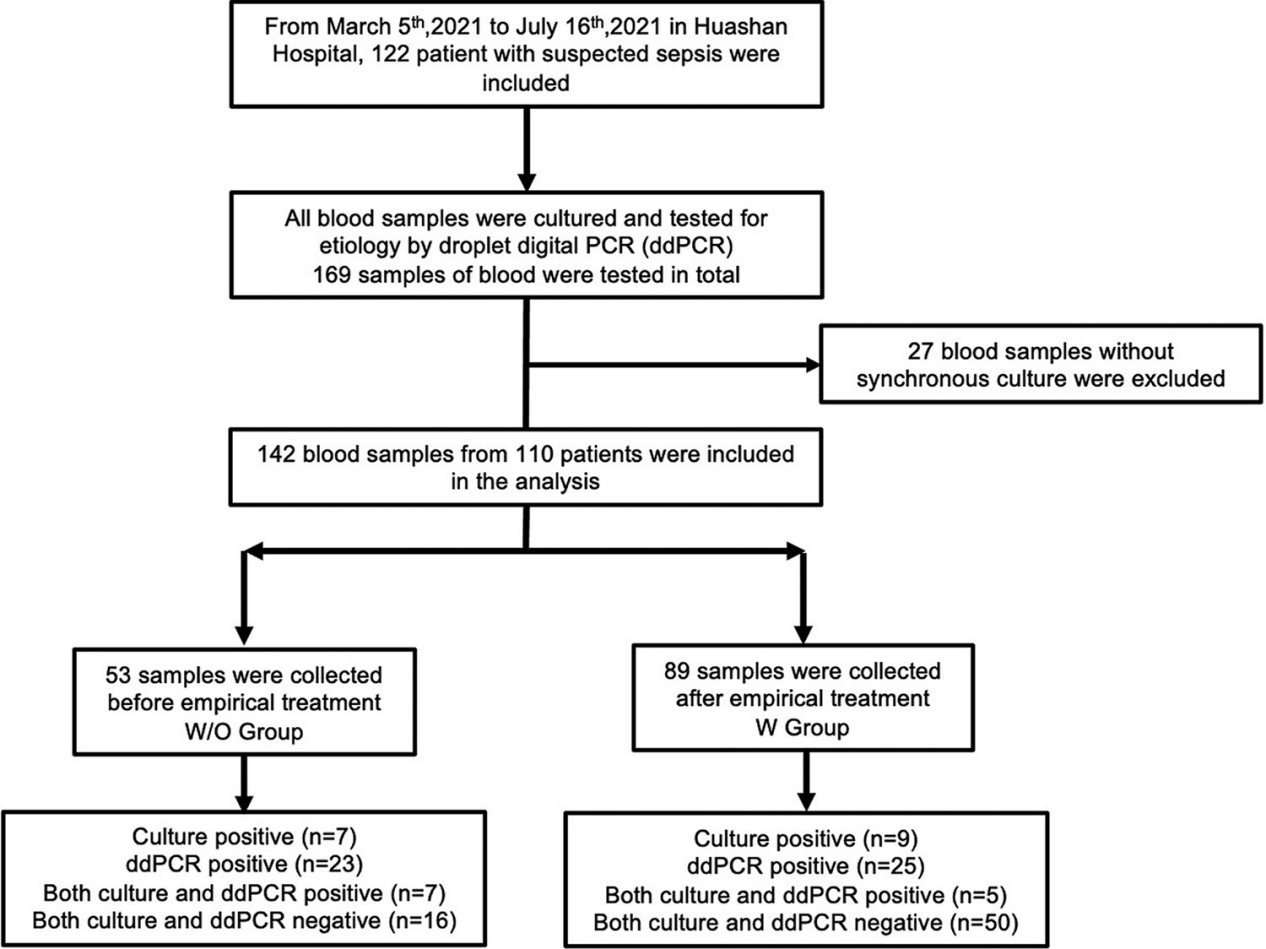
Flowchart of enrollment. W/O group: patients did not undergo antimicrobial treatment, or the treatment duration was less than 3 days. W group: patients accepted 3 days or more of continuous antimicrobial treatment.

In the study, 122 patients were enrolled for eligibility, 29 of whom had repeated ddPCR tests up to 7 times during the progression of disease, and most of them were from the department of infectious disease (75.5%). A total of 169 blood samples were collected for ddPCR detection. In order to evaluate the clinical performance of ddPCR compared to culture we excluded 27 blood samples for no synchronous blood culture (Fig. 1). Among the remaining 142 blood samples from 110 patients, 53 were from patients without antimicrobial treatment within 3 days before collection (W/O group), 89 were from patients who had received antimicrobial treatment for 3 days or more (W group). The baseline characteristics of the two groups are listed in Table 1. Factors including gender, age, body mass index (BMI), physical examination findings, and blood laboratory tests were mostly equally distributed among two groups.

**TABLE 1:** General characteristics (*N* = 110 patients)^*a*^

Characteristic	Value
Age (yrs)	52.9 ± 18.2
Male [no. (%)]	63 (57.3)
BMI	22.2 ± 3.7
SIRS [no. (%)]	87 (79.1)
qSOFA ≥ 2 [no. (%)]	24 (21.8)
Physical examination findings	
Body temp (^o^C)	37.9 ± 1.2
Heart rate (BPM)	98.3 ± 18.7
Systolic blood pressure (mmHg)	118.7 ± 20.2
Diastolic blood pressure (mmHg)	73.0 ± 12.5
Change of consciousness [no. (%)]	23 (20.9)
Blood laboratory examination	
White blood cell count (× 10^9^/L)	8.9 ± 6.1
Neutrophil (%)	73.9 ± 20.7
Platelet count (× 10^9^/L)	119.0 ± 134.2
C reactive protein (mg/L)	103.7 ± 80.5
Procalcitonin (ng/mL)	5.52 ± 12.49
Serum creatinine (μmol/L)	77.84 ± 62.79
Total bilirubin (μmol/L)	30.32 ± 79.8
Fibrinogen, mg/dL	4.99 ± 4.3
Risk factors	
Usage of corticoids in the past mo. [no. (%)]	31 (28.2)
Usage of immune-suppressive drugs in the past mo. [no. (%)]	9 (8.2)
Mechanical ventilation [no. (%)]	14 (12.7)
Clinical characteristics during hospitalization	
Renal replacement therapy [no. (%)]	4 (3.6)
Usage of vasoactive drugs [no. (%)]	18 (16.4)
Ward	
Department of Infectious Diseases [no. (%)]	83 (75.5)
Intensive care units [no. (%)]	4 (3.6)
Emergency Department [no. (%)]	8 (7.3)
Other wards [no. (%)]	15 (13.6)

a Continuous variables (mean ± SD). W/O group, patients did not undergo antimicrobial treatment, or the treatment duration was less than 3 days. W group, patients received three days or more of continuous antimicrobial treatment. BMI, body mass index; SIRS, systemic inflammatory response syndrome; qSOFA, quick sequential organ failure assessment; ddPCR, droplet digital PCR; BPM, beats per minute.

### Overall diagnostic performance of ddPCR.

**FIG 2:**
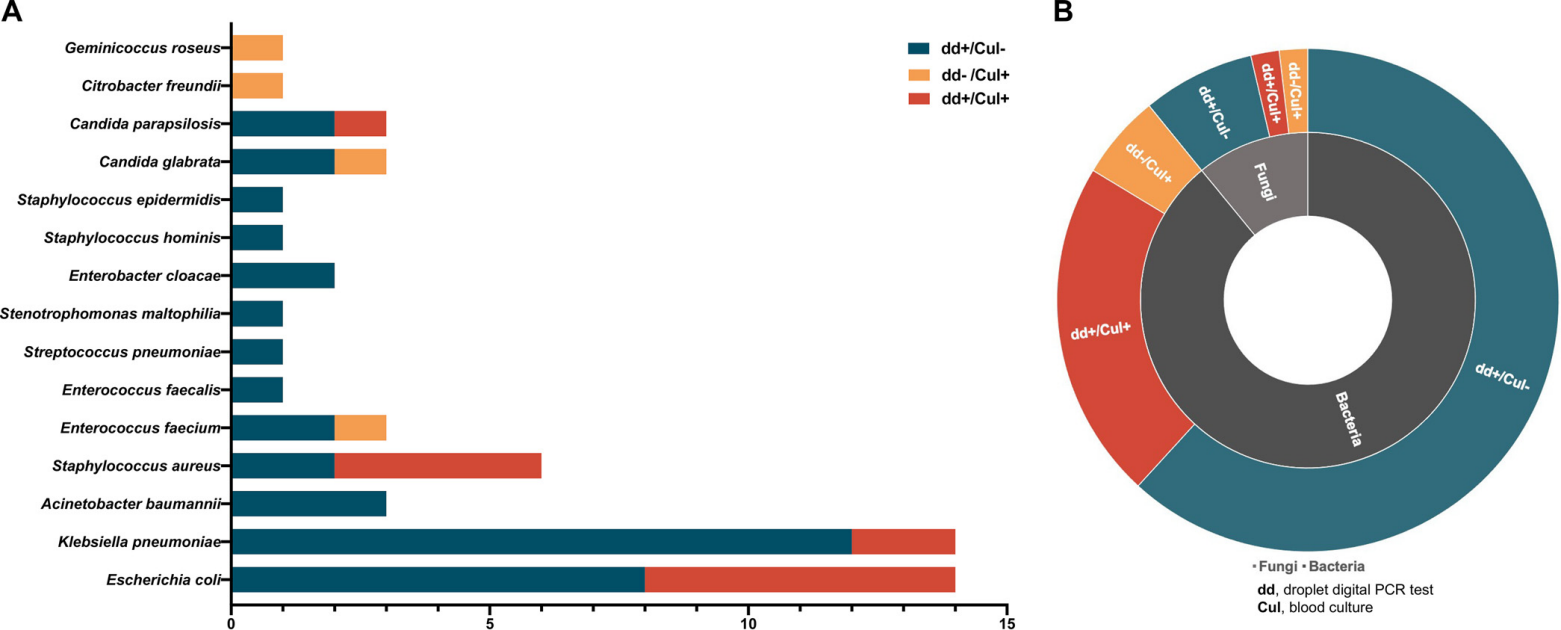
Distribution of detected pathogens by ddPCR and culture. (A) Pathogens detected by droplet digital PCR (ddPCR) and blood culture. Green bars represent the cases in which the pathogens were detected only by ddPCR and the blood culture reported negative. Yellow bars mean that the pathogens were detected by blood culture only, while the red bars mean the pathogens were detected both by blood culture and ddPCR. The length of the bar represents the number of cases. (B) In the inner ring, light gray represents fungi and dark gray represents bacteria. In the outer ring, red indicates that results of both ddPCR and culture were positive. Yellow means only culture was positive. Green means only ddPCR was positive. The size of the ring indicates the number of cases. dd+, positive results of droplet digital PCR test; dd–, negative results of droplet digital PCR test; Cul+, positive results of blood culture; Cul–, negative results of blood culture.

In general, etiological diagnosis showed that blood culture reported 16 positive results, and ddPCR detection showed 43 positive results from 142 blood samples in total. Klebsiella pneumoniae and Escherichia coli were the most commonly identified pathogens (Fig. 2A and B). To analyze the diagnostic performance of ddPCR compared to blood culture two cases of pathogens cultured from blood samples that were outside the ddPCR detection spectrum were excluded, Geminicoccus roseus and Citrobacter freundii. Therefore, there were 14 positive blood culture results from 140 samples in total. Overall, ddPCR reported a 75.00% (12/16) (95% CI, 47.41 to 91.67%) sensitivity compared to culture in patients with suspected BSI. In addition, the specificity was 75.40% (95/126) (95% CI, 66.78 to 82.44%) compared to blood culture (Table 2). Moreover, ddPCR showed a relatively higher detection rate than that of culture (30.28% versus 11.27%, Pearson chi-square = 15.596; *P* < 0.0001), as its extradetection rate was up to 19.01% (27/142) (95% CI, 13.11 to 26.63%), and 74.19% (23/31) of the cases with negative culture results and positive ddPCR reports were in accordance with the clinical diagnosis.

**TABLE 2:** Sensitivity, specificity, and extradetection rate^*a*^

Test	Sensitivity compared to culture			Specificity compared to culture (%) (95% CI)	Extra detection rate compared to culture (%) (95% CI)
	Without empirical treatment (%) (95% CI)	With empirical treatment (%) (95% CI)	Overall (%) (95% CI)		
ddPCR	100.00 (7/7) (56.09–100.00)	55.56 (5/9) (22.66–84.66)	75.00 (12/16) (47.41–91.67)	75.40 (95/126) (66.78–82.44)	19.01 (27/142) (13.11–26.63)
Culture			100 (16/16) (75.93–100.00)		

a ddPCR, droplet digital PCR. 95% CI, 95% confidence interval.

In the W group, the detection rate of blood culture was 10.11% (9/89) (95% CI, 5.01 to 18.79%), while the ddPCR detection rate was up to 24.72% (22/89) (95% CI, 16.46 to 35.20%), which was significantly higher than that of culture (*P* = 0.010, Pearson Chi-square = 6.601). Additionally, the sensitivity of ddPCR was 55.56% (5/9) (95% CI, 22.66 to 84.66%) compared to culture in the empirically treated patients, and the specificity was 78.75% (63/80) (95% CI, 67.89 to 86.79%). In the W/O group, the detection rate of culture was 13.21% (7/53) (95% CI, 5.92 to 25.96%), while the ddPCR detection rate was 39.62% (21/53) (95% CI, 26.76 to 53.98%). Moreover, compared to culture, the sensitivity of ddPCR was 100% (7/7) (95% CI, 56.09 to 100.00%), and the specificity was 69.57% (32/46) (95% CI, 54.08 to 81.81%). The effect of empirical treatment on the detection rate of both culture and ddPCR was not significant (10.11% versus 13.21% and 24.72% versus 39.62%, respectively; *P* > 0.05), and the sensitivity and specificity of the ddPCR test were both affected by the use of antibiotics (71.43% versus 100% and 78.75% versus 69.57%, respectively; *P* > 0.05).

**FIG 3:**
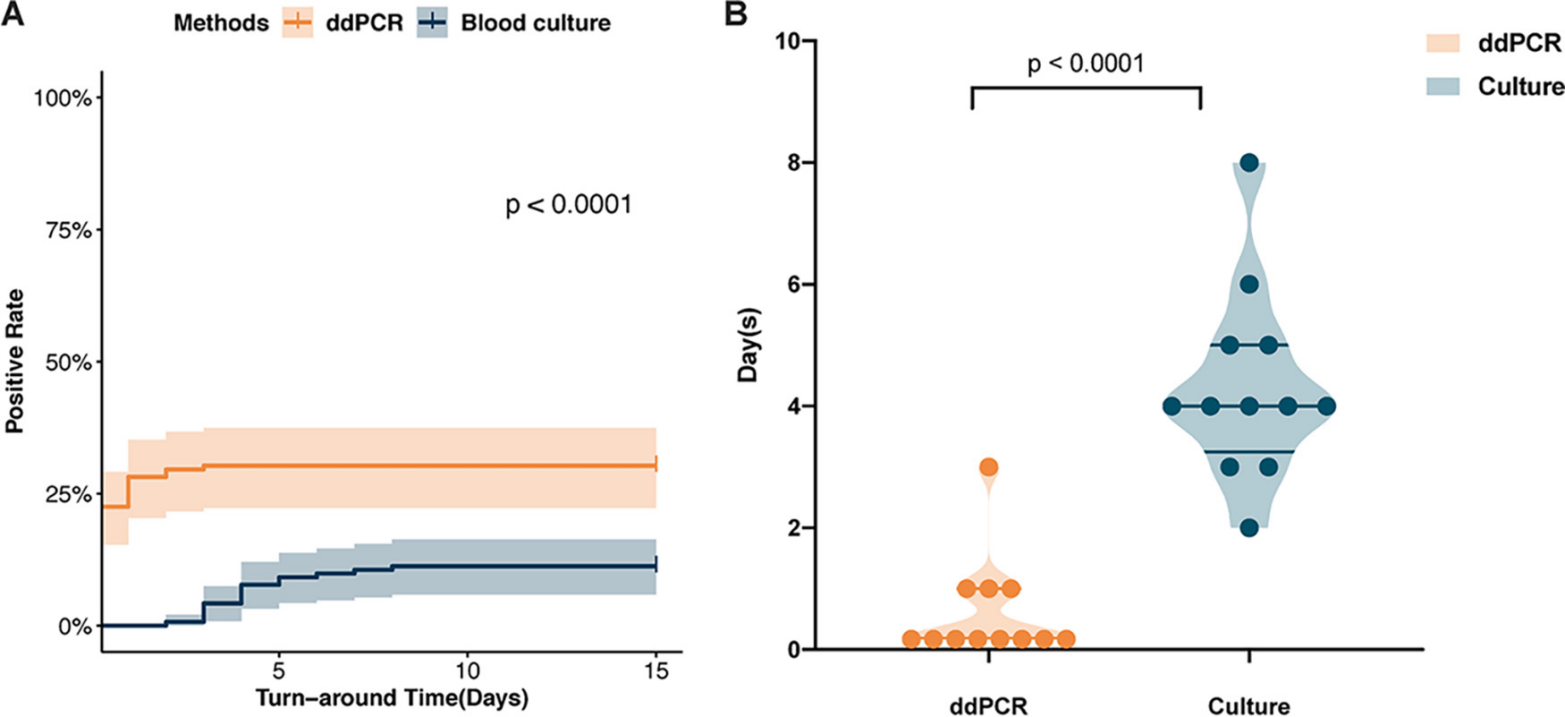
Comparison of turnaround times (TAT) between ddPCR and culture. (A) Comparison of positive rate and TAT between ddPCR and blood culture among all the participants. (B) Comparison of TAT between ddPCR and culture among the patients with positive results from both methods. ddPCR, droplet digital PCR; culture, blood culture.

We further analyzed the TAT required to determine the pathogenic diagnosis. For blood culture, the mean of the time required for report was 4.3 days, while the ddPCR test required significantly less time (0.46 day) to identify the pathogens (*P* < 0.0001) (Fig. 3A and B).

### Correlative analysis between ddPCR and laboratory results of inflammatory indicators.

**FIG 4:**
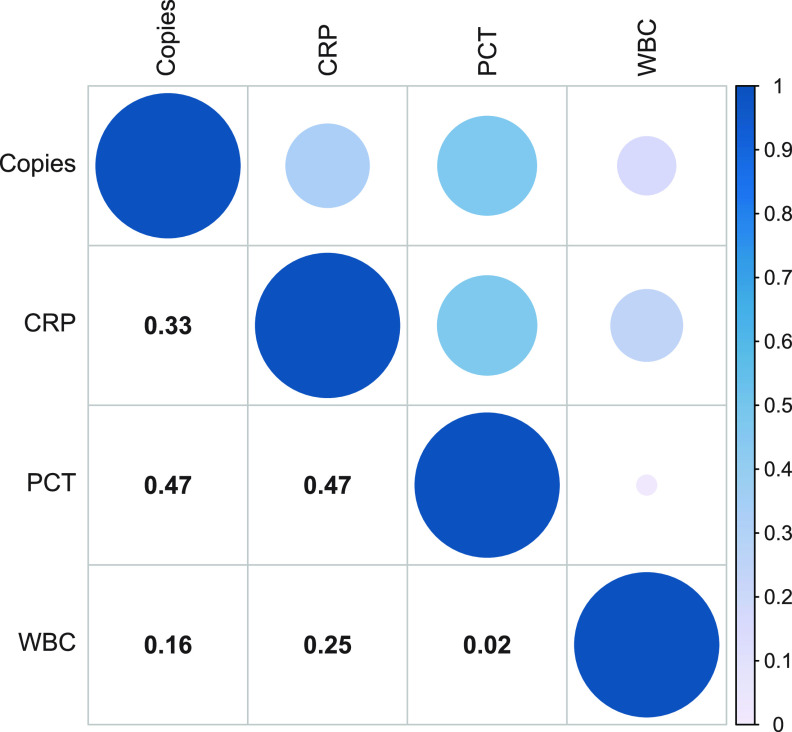
Correlations Between copies of pathogens detected by ddPCR and CRP, PCT, and WBC. The number in the box is Pearson’s correlation coefficient (*r*). The larger the circle and the darker the color, the greater is the *r* value. ddPCR, droplet digital PCR; CRP, C-reactive protein; PCT, procalcitonin; WBC, white blood cells.

In general, we calculated the correlation between the clinal inflammatory indicators (CRP, PCT, and WBC) and the pathogen copy value detected by ddPCR in the blood. We found that the CRP had a positive correlation with the pathogen loads detected by ddPCR (Spearman’s rho = 0.333, *P* = 0.027), and PCT had a significant positive correlation with the ddPCR results (Spearman’s rho = 0.469, *P* = 0.001) (Fig. 4). However, the WBC value was shown to be less correlative with the pathogen loads detected by ddPCR (Spearman’s rho = 0.162, *P* = 0.294).

**FIG 5:**
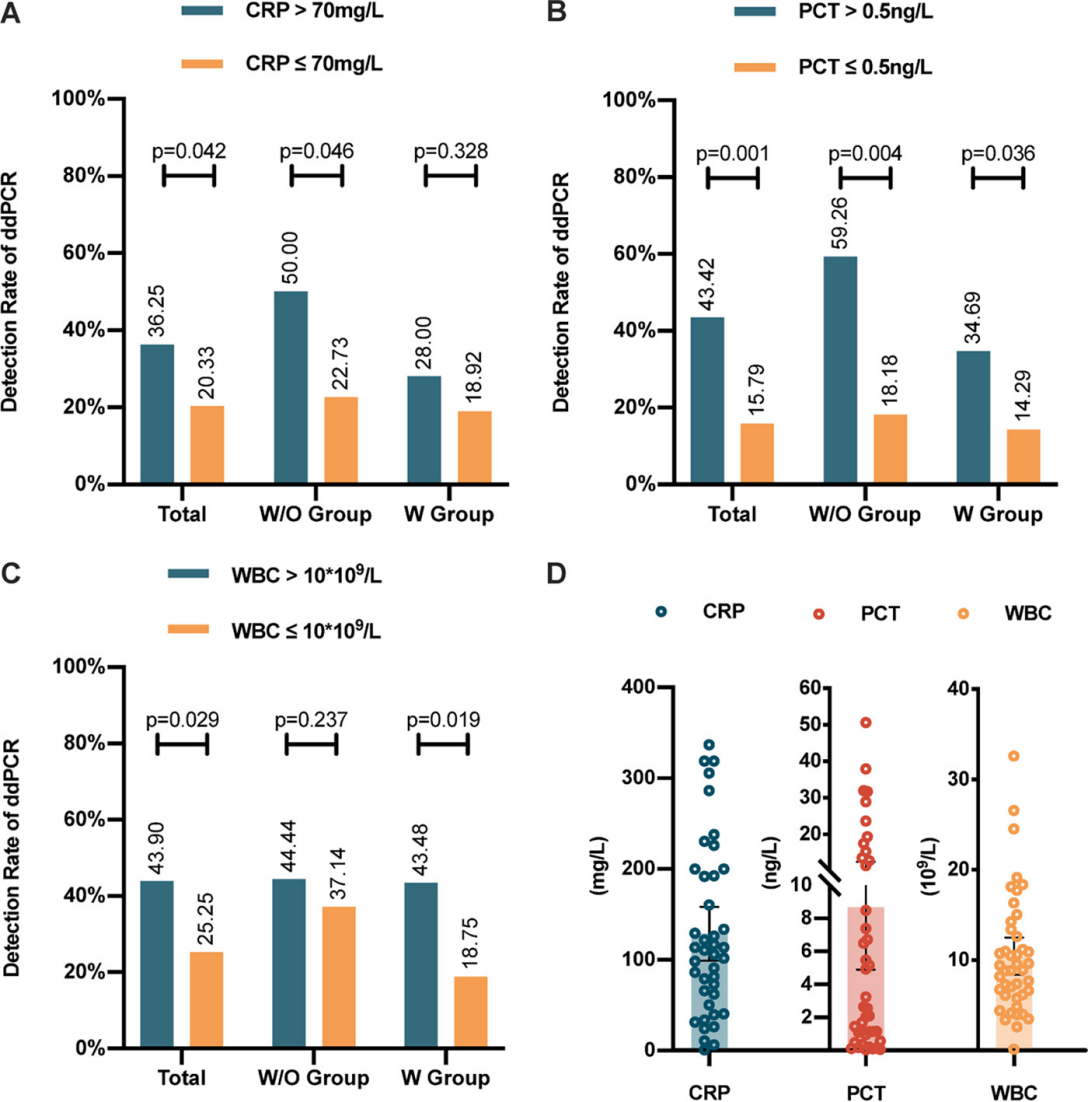
The influence of CRP, WBC, and PCT on the detection rate of ddPCR in BSI. (A to C) The influence of CRP, PCT, and WBC on the detection rate of ddPCR in bloodstream infections with or without previous anti-infective therapy. (D) The distribution of participants’ CRP, PCT, and WBC values. W/O group, patients did not undergo antimicrobial treatment, or the treatment duration was less than 3 days. W group, patients received 3 days or more of continuous antimicrobial treatment. ddPCR, droplet digital PCR; BSI, bloodstream infection; CRP, C-reactive protein; PCT, procalcitonin; WBC, white blood cells.

Further assessment of the influence of blood laboratory examinations on ddPCR detection rate showed that, in BSI, the detection rate of ddPCR was significantly higher in patients with a CRP value of >70 mg/L (χ^2^ = 4.134, *P* = 0.042) (Fig. 5A), a PCT value of >0.5 ng/L (χ^2^ = 11.510, *P* = 0.001) (Fig. 5B), or a WBC count of >10 · 10^9^/L (χ^2^ = 4.739, *P* = 0.029) (Fig. 5C). We found that the more severe the inflammation caused by sepsis, the higher was the possibility of a positive ddPCR result. In a subgroup analysis of patients without empirical antibiotic treatment (W/O group), a CRP value of >70 mg/L (χ^2^ = 3.989, *P* = 0.046) or a PCT value of >0.5 ng/L (χ^2^ = 8.468, *P* = 0.004) was also related to a higher ddPCR detection rate, while in the W group, a PCT value of >0.5 ng/L (χ^2^ = 4.399, *P* = 0.036) or a WBC count of >10 · 10^9^/L (χ^2^ = 5.476, *P* = 0.019) suggested a higher positive rate of ddPCR (Fig. 5).

### Quantitative value of ddPCR in the dynamic surveillance of BSI.

**FIG 6:**
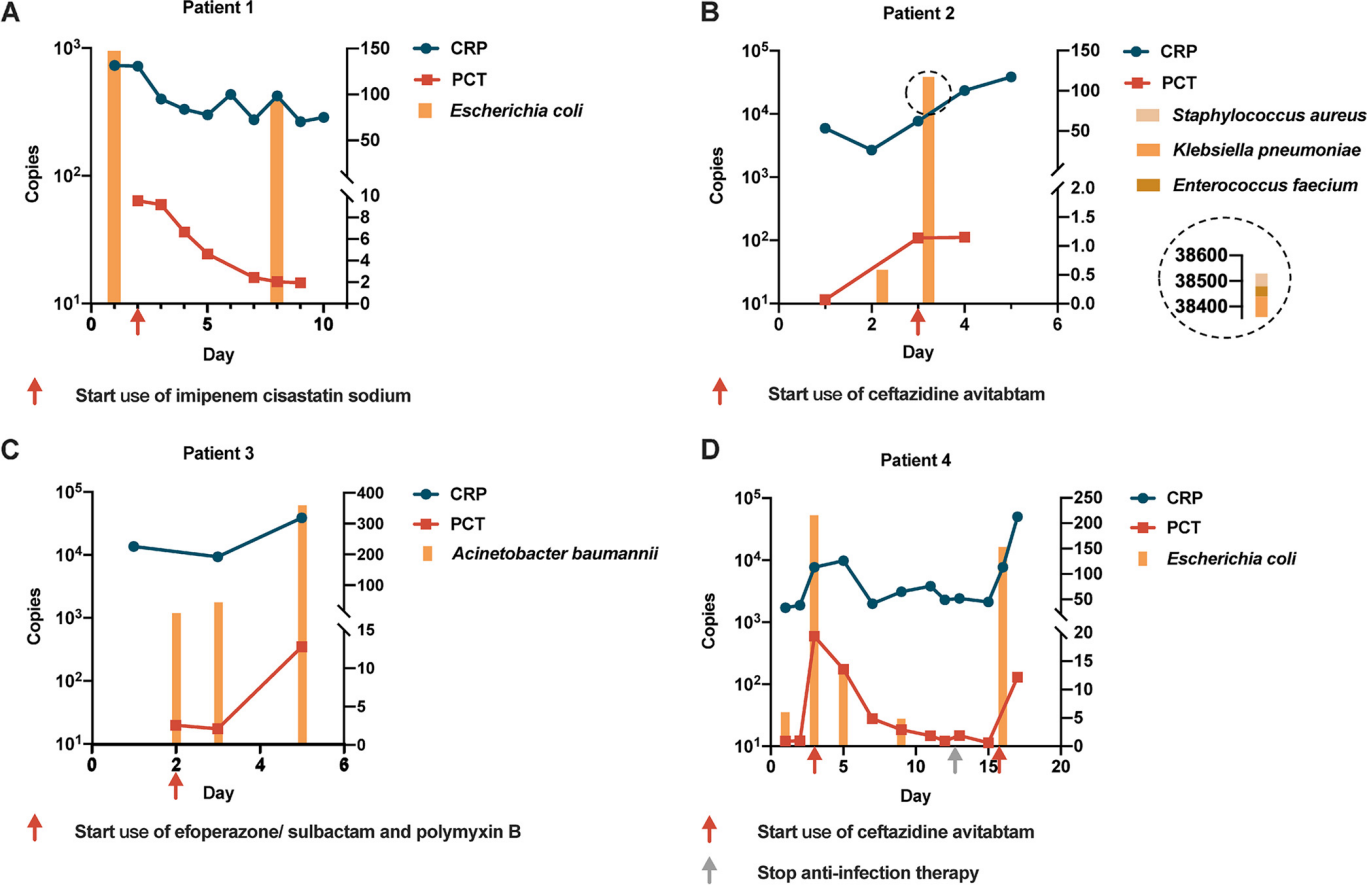
The quantitative value of ddPCR in the dynamic surveillance of suspected bloodstream infections. The copy value reported by droplet digital PCR (ddPCR) was in accordance with synchronous CRP and PCT values. The red and gray arrows indicate the time points when the anti-infective treatment was started and stopped, respectively. CRP, C-reactive protein; PCT, procalcitonin.

We performed repeated ddPCR tests on four cases to observe the dynamic surveillance role of ddPCR during BSI. The results showed direct correlations between ddPCR quantitative copies and WBC, PCT, and CRP values (Fig. 4). In the four patients (Fig. 6) with multiple consecutive WBC, PCT, and CRP results, it can be seen that their change directions were similar. Notably, when patients received effective antimicrobial treatment (Fig. 6A and D), ddPCR sequencing reads declined, which was consistent with synchronously decreased WBC, PCT, and CRP values.

### Clinical application potential of ddPCR in antimicrobial therapy.

**FIG 7:**
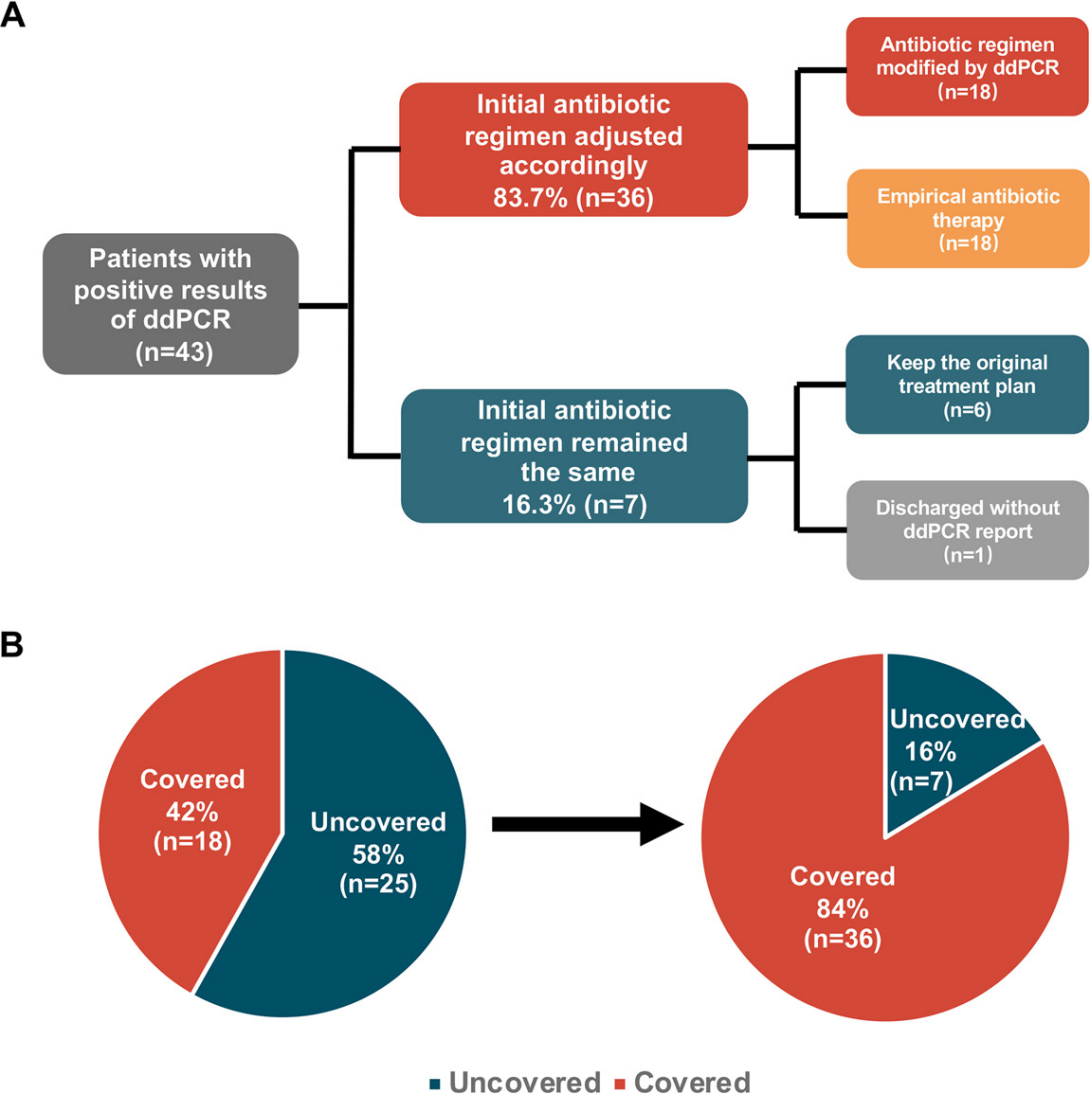
Changes in antibiotic treatment regimen due to intervention with ddPCR. (A) Antibiotic regimen adjustment in patients with positive results of droplet digital PCR (ddPCR). (B) According to the results of ddPCR, medication regimens were adjusted, and pathogens were covered in more cases.

We screened out ddPCR-positive cases (*n* = 43) and divided them into two groups based on their antimicrobial regimen adjustment: those antimicrobial regimens adjusted after the report of the ddPCR result (intervene group, *n* = 22) and those with a continued previous antimicrobial plan (continue group, *n* = 21). Overall, the proportion of patients with an optimized antimicrobial regimen was up to 51.2% (22/43) in our study; 81.8% (18/22) of them had an antimicrobial regimen started or altered as shown in the Fig. 7A, and one patient (4.5%, 1/22) had no rank change but merely switched antibiotics. The other three of them (13.6%, 3/22) had deescalation adjustment.

We further calculated the causative pathogen coverage rate (CPCR). For 40% (17/43) of patients with positive ddPCR results, the empirical antimicrobial therapy they initially received was proved to cover the causative pathogens, and the regimen was kept the same. In addition, 40% (17/43) of the patients changed antimicrobial medication plans according to the ddPCR results. Collectively, for patients with positive ddPCR results, the CPCR was increased from 40% (17/43) to 81% (35/43) (Fig. 7B). For the eight uncovered samples, two patients were discharged before the ddPCR results became available. Only 14% (6/43) of the results were considered unreliable (diagnosis unsupported) or not significant enough to induce treatment adjustment at the time; thus, the treatment plan was kept the same.

Out of 12 cases with both digital PCR and blood culture yielding positive results, further evaluation showed that a therapy adjustment was deemed necessary in 5 (intervene group), and the pathogen coverage was increased from 25% (3/12) to 67% (8/12) (see Table S3 in the supplemental material). Two patients were discharged before the digital PCR result became available, and two patients had continued therapy because the digital PCR results were considered implausible at the time.

## DISCUSSION

In our study, ddPCR detected a wide range of pathogens, covering bacteria and fungi that commonly cause bloodstream infections, such as Staphylococcus aureus. We evaluated the diagnostic performance of ddPCR and compared it with that of blood culture. Overall, the additional detection rate of ddPCR was as high as 19.01% (95% CI, 13.11 to 26.63%), and 10 bacteria and fungi were detected in 31 cases with negative blood cultures.

In previous studies, several culture-independent molecular techniques have been reported with the ability of rapid detection of pathogens in whole blood (19, 20). PCR-based assays were widely known, such as the MagicPlex sepsis test based on multiplex real-time PCR (Seegene, Seoul, South Korea). Although the results of the MagicPlex sepsis test can be obtained within 3 to 5 h, its sensitivity is relatively low, ranging from 37 to 65% (21). SepsiTest (Molzym, Bremen, Germany), based on broad-range PCR, can detect 345 bacteria and fungi. However, it takes 8 to 12 h to get the results due to its complicated steps, and its sensitivity fluctuates greatly (21 to 85%), showing its unstable quality control (6, 10, 22, 23). In addition, the PCR-based PLEX-ID (Abbott Molecular, Carlsbad, CA, USA) is a universally accepted molecular device, with its ability of detecting a wide range of pathogens (up to 800) and high sensitivity (83%). However, the high sensitivity and wide range of pathogen detection are dependent on the association of microbe detection by PCR and amplicon analysis by electrospray ionization mass spectrometry (ESI-MS); thus, the PLEX-ID machine costs around $200,000, causing this method to not be widely used (6, 21, 24–26).

In this study, compared with blood culture, ddPCR had a relatively high sensitivity of 85.71% (95% CI, 56.15 to 97.48%), a specificity of 71.43% (95% CI, 30.26 to 94.89%), and a satisfactory extra detection rate of 19.01% (95% CI, 13.11 to 26.63%). At the same time, ddPCR showed its great advantage over traditional blood culture in the time required for testing. The average TAT was 0.46 day (around 11 h). Moreover, if we exclude the actual transport time of clinical specimens, the actual time ddPCR requires is around 3 h. Therefore, the rapid and precise characteristics of ddPCR show its potential to improve clinical decision making and the possible benefit of reducing BSI mortality and disability rates.

In addition, ddPCR provided accurate and quantitative load data of causative pathogen in a timely manner, partially reflecting the severity of the infection and inflammation. Therefore, ddPCR has the potential for real-time monitoring the patient’s condition and for providing early warning for clinicians (16). The ddPCR detection gives feedback on loads of specific pathogens as well as the common antibiotic-resistant genes, providing important information for clinical decision making regarding antibiotic usage. Furthermore, the overall sensitivity and specificity of ddPCR detection is less affected by empirical medication, thus making ddPCR stable and convincing in clinical application.

Another interesting finding revealed that the ddPCR-positive group had a significantly higher WBC level, CRP level, and PCT value than ddPCR negative group. This could be explained by more severe inflammation in sepsis patients usually indicating the existence of larger loads of microorganisms, which is more likely to lead to a positive ddPCR report. An increasing load of pathogen could then further stimulate a series of immune inflammatory responses, and thus, it was not difficult to understand that the pathogen loads detected by ddPCR were positively correlated with the measured inflammatory indicators. Further cutoff value analysis found that patients with a CRP level of >70 mg/L, a PCT value of >0.5 ng/L, or a WBC count of >10 · 10^9^/L would have a significantly higher ddPCR detection rate, implying that these patients may be more likely to benefit from ddPCR. To validate our assumptions, we found direct correlations between ddPCR pathogen loads and blood WBC, CRP, and PCT levels (Fig. 4), further proving that the changes of pathogen load detected by ddPCR were in accordance with synchronous blood WBC, CRP, and PCT levels, particularly relevant to PCT, an inflammatory index commonly used clinically to judge sepsis. It could be explained that the pathogen load was often closely related to the severity of BSI (27–29). Since pathogens stimulate a series of inflammatory responses whose intensities are reflected by inflammatory indicators (30, 31), it was not difficult to understand that the pathogen loads detected by ddPCR were positively correlated with the measured value of inflammatory indicators.

When assessing the potential impact of ddPCR on the antimicrobial therapy in BSI, we found that 22 out of 43 (51.2%) ddPCR-positive cases had an adjustment of antimicrobial therapy, which is similar to those (46% and 39%) reported in previous studies (10). In 18 out of 22 cases (81.8%), there was an antimicrobial therapy escalation as a result of microbiological findings. The major factor contributing to this high percentage was that 77.8% (14/18) of escalation cases were from patients without antimicrobial treatment 3 days before sampling (W/O group), thus starting an antimicrobial regimen. It can be speculated that, given an mRDT TAT as short as 4 h, some clinician would wait for the results of ddPCR to start antimicrobial therapy, which could cause potential harm to patients because of delayed initiation of antimicrobial treatment, especially patients suffering from septic shock. One of the limitations of our approach is that antimicrobial treatment therapy changes were analyzed *post hoc*, which could lead to mal-interpreted results compared to those during actual clinical courses. Also, since this is not a randomized controlled trial, whether therapeutic adjustment as a result of digital PCR testing has an impact on the prognosis of patients with suspected BSIs could not be determined. Furthermore, since this is a study conducted in a single center, bias such as clinical decision-making preferences is inevitable (8, 32).

However, ddPCR also has inevitable shortcomings. First, though studies have shown that the detection rate of ddPCR is significantly higher than that of mNGS within the detection range of ddPCR targets, ddPCR can only detect target pathogens with existing probes. Therefore, the ddPCR detection scope of pathogens is not as wide as that of mNGS. Second, in this study, there were two cases of positive blood culture reporting of pathogens beyond the scope of the ddPCR test. One was *Citrobacter* and the other was gemini measles. Although these bacteria do not commonly cause bloodstream infections according to The China Antimicrobial Surveillance Network (CHINET) (http://www.chinets.com), this revealed the limitation of ddPCR in pathogen detection due to the panel design. Third, the study was conducted without a control group, as in other previous studies on diagnostic performance evaluation (33, 34), and all the patients were enrolled at the tertiary care center; thus, the study population were less representative than the all-encompassing one. Fourth, the antimicrobial treatment therapy changes were analyzed retrospectively, which could lead to mal-interpreted results. Furthermore, the application of ddPCR still relied heavily on manual labor; in the current situation, the whole process of ddPCR basically required manual participation. This led to a longer turn-around-time, especially for specimens collected during non-work hours. The highly efficient automatic machines are expected to shorten the time to results and improve the detection stability. Also, in this single-center prospective study, whether therapeutic adjustment as a result of digital PCR test had an impact on the prognosis of patients with BSI could not be determined, and more studies are still needed in the future.

**Conclusion.** This single-center study demonstrated that in suspected BSI patients, ddPCR had an overall superior detection rate of potential pathogens compared to blood culture. For patients who had received empirical antibiotic treatment, ddPCR had significant diagnostic advantages over blood culture; however, the detection rate and the sensitivity of ddPCR decreased. Our findings also showed that elevated WBC, PCT, and CRP values are correlated with higher copy numbers of pathogens detected by ddPCR in BSI. Furthermore, ddPCR can dynamically surveil pathogen loads and disease progression using quantitative value analysis. The clinical impact of ddPCR is of significance since early detection of pathogens could improve the antimicrobial regimens and guide precision treatment therapy. In the future, multicenter studies will be needed to further explore the clinical benefits for patient prognosis using ddPCR in suspected BSI infections.

## MATERIALS AND METHODS

### Setting and data collection.

This single-center prospective cohort study with no control group was carried out at Huashan Hospital, a tertiary center in Shanghai, China. All the data for this study were collected from the electronic medical records system of Huashan Hospital. The protocol for the conduct of this study was reviewed and approved by Huashan Hospital ethical committee, and the patients or their surrogates signed informed consent.

### Patients and samples.

Patients older than 18 years with suspected bloodstream infection were eligible for inclusion if they were admitted to Huashan Hospital between March 2021 and July 2021. The participant inclusion criteria include age ≥18 years and suspected BSI with at least two of the following clinical manifestations or laboratory tests results: (i) peak body temperature exceeding 38.3°C, (ii) an increased WBC count which was hard to explain with noninfectious factors, (iii) increased CRP/erythrocyte sedimentation rate (ESR)/ferritin (FER) values based on clinician judgement, (iv) elevated PCT value that was difficult to explain with noninfectious factors, and (v) risk factors for BSI. Patients records in the electronic medical records system were collected with written informed consent. Synchronous blood samples, defined as blood specimens collected within 24 h, were sent for ddPCR tests and culture according to clinical needs, as well as conventional tests including CRP, PCT test, and routine blood examination. The date of the synchronous blood samples collected for ddPCR test and patients’ clinical and laboratory data were recorded. Enrollment and exclusion criteria are listed in Table 3 and in Table S1. The laboratory flow of ddPCR is shown in Fig. S1.

**TABLE 3:** Enrollment and exclusion criteria^*a*^

Category	Criteria
Enrollment criteria	Age ≥18 yrs
	Suspected BSI (at least two of the following clinical manifestations or laboratory test results should be met): 1. Peak body temp exceeding 38.3°C 2. Increased WBC count which was hard to explained by noninfectious factors 3. Increased CRP/ESR/FER values that were hard to explained by noninfectious factors 4. Elevated PCT value that was difficult to explained by noninfectious factors 5. Risk factors for BSI^*b*^
	Patients with records in the electronic medical records system at Huashan Hospital
Exclusion criteria (must all be met)	Age <18 yrs
	Patient did not meet the criteria of suspected BSI
	No patient records in the electronic medical records system at Huashan Hospital
	Patient refused to draw blood or participate in this study
	Patient participating in other clinical studies

a BSI, bloodstream infection; WBC, white blood cells; CRP, C-reactive protein; ESR, erythrocyte sedimentation rate; FER, ferritin; PCT, procalcitonin; ICU, intensive care unit.

b Risk factors of BSI: (i) advanced age (≥65 years) (ii) Immunosuppression. Immunosuppressant medications including corticoids used in the past month and comorbidities that depress host defense (e.g., neoplasms, especially cancer, renal failure, hepatic failure, AIDS, asplenism). Also, diabetes and obesity were taken into consideration since they may alter the immune system, resulting in an elevated risk for developing BSIs. (iii) Previously diagnosed or family history of immunodeficiency. (iv) Intensive care unit admission. Approximately 50% of ICU patients have a nosocomial infection. (v) Invasive medical procedures such as mechanical ventilation and intravascular catheter placement.

### Outcomes.

The primary endpoint of this study was assessment of ddPCR diagnostic performance compared with blood culture, and the secondary endpoint was the application value of ddPCR in suspected BSI patients in clinical practice. Also, the clinicians’ decisions were mainly made based on the combination of examinations such as blood culture and other laboratory and clinical cues, other than ddPCR; we compared the ddPCR results to the clinicians as well. The impact was analyzed by retrospective review of clinical records when the diagnostic results and antimicrobial therapy regimens were available. We classified antimicrobials according to previously established ranking categories (35).

### Sample preparation and data analysis.

Nucleic acid was extracted from the blood samples. First, peripheral blood specimens were centrifuged at 1,600 × *g* for 15 min at 4°C. Second, 2 mL of supernatant plus 10 μL of internal control was transferred to an Auto-Pure 10B nucleic acid purification system (Hangzhou Allsheng Instruments Co., Ltd., Hangzhou, China) for cell-free DNA (cfDNA) isolation using a magnetic serum/plasma DNA *K*it (Tiangen Biotech, Beijing, China) according to the manufacturers’ instructions. Then the cfDNA was eluted into 60 μL of 10 mM Tris-EDTA buffer (a solvent that can dissolve DNA and RNA and protect them from degradation) and stored at −80°C until final analysis.

Droplet digital PCR (ddPCR) analysis was performed using a 5-fluorescent-channel droplet digital PCR system (Pilot Gene Technology [Hangzhou] Co., Ltd., Hangzhou, China). The targeting pathogens from PilotBac, PilotFungi, and PilotAMR assay panels are provided in Table S2. Briefly, 5 μL of cfDNA template was added to 10 μL of the ddPCR premix, which includes detection primers, probes, and the necessary components for PCR amplification. The reaction mixture was gently mixed and added into a ready-to-use disposable plastic chip. About 20,000 water-in oil emulsion droplets were generated inside the chip by a droplet generator (DG32, Pilot Gene Technologies). Chips were then amplified in a thermal cycler (TC1, Pilot Gene Technologies) using the following cycling parameters: 95°C for 5 min, followed by 40 cycles at 95°C for 15 s and 60°C for 60 s. Finally, chips were loaded into a chip scanner (iScanner 5, Pilot Gene Technologies) for fluorescence signal reading and further data analysis. The data were analyzed with GeneDPT software (Pilot Gene Technologies). According to the manufacturers’ instructions of the assay panels, the threshold for target detection was 0.7 copy/μL. A ddPCR is defined as positive if the concentration is over the threshold.

### Diagnostic assessment of ddPCR.

The diagnostic performance of the ddPCR was assessed through the following steps. First, we counted the total pathogens detected by ddPCR. Second, we compared the sensitivity of ddPCR to blood culture; ddPCR sensitivity compared to culture = ddPCR-positive/culture-positive. Third, the specificity compared to blood culture was statistically evaluated; specificity compared to culture = ddPCR-negative/culture-negative. In addition, we calculated the detection rate of ddPCR as well as the additional detection rate of it compared to conventional blood culture; namely, ddPCR additional detection rate = (ddPCR-positive – culture-positive)/overall-samples. Then, we divided the patients into two groups, the W group and the W/O group, according to the antimicrobial treatment they received. Patients in the W group experienced antimicrobial treatment sequentially for 3 days or more before ddPCR and blood culture. Patients that did not receive any antimicrobial treatment, or whose treatment was not sequential (fewer than 3 days) before ddPCR and blood culture were included in the W/O group. To further analyze the clinical application value of ddPCR in rapid pathogen detection, we defined the time from specimen sampling to result reporting as the turnaround time (TAT).

### Antimicrobial treatment assessment.

To analyze the impact of the digital PCR results on antimicrobial treatment, we retrospectively reviewed the clinical records and analyzed them, when the diagnostic results and antimicrobial regimens were available. We classified antimicrobials according to a previously reported ranking schema (35). According to the schema, antibiotics were ranked into 4 categories: narrow spectrum, broad spectrum, extended spectrum, and agents targeted for protection. An electronic deescalation can be defined as reduction in either the number of antibiotics or category rank. We defined “pathogen covered” as the antimicrobial regimens being effective for treating causative pathogens detected by ddPCR or blood culture, to calculate the causative pathogen coverage rate (CPCR) (36).

### Statistics analysis.

For general characteristics and laboratory tests, continuous variants were described by means when they conformed to the Kolmogorov-Smirnov test and by medians when they did not. Chi-square tests were used to evaluate independent binomial variables, and a *P* value of <0.05 was considered significant. We analyzed Mann-Whitney test results to compare differences across ddPCR subgroups. Statistical analyses were conducted and figures were created using SPSS 26.0 (IBM Corp., Armonk, NY, USA), Prism 8.4.0 software (GraphPad Software, San Diego, CA, USA), and RStudio 4.0.3 software (RStudio Software, Boston, MA, USA).

### Ethics approval and consent to participate.

The study was approved by the Huashan Hospital ethical committee, and the methods were carried out in accordance with the approved guidelines of the institution.
