# Correction for Lin et al., “Clinical Diagnostic Performance of Droplet Digital PCR for Suspected Bloodstream Infections”

**DOI:** 10.1128/spectrum.01534-23

**Published:** 2023-12-15

**Authors:** Ke Lin, Yuanhan Zhao, Bin Xu, Shenglei Yu, Zhangfan Fu, Yi Zhang, Hongyu Wang, Jieyu Song, Mingxiang Fan, Yang Zhou, Jingwen Ai, Chao Qiu, Haocheng Zhang, Wenhong Zhang

## AUTHOR CORRECTION

Volume 11, no. 1, e01378-22, 2023, https://doi.org/10.1128/spectrum.01378-22.

Page 1, abstract, lines 8 to 12: “In BSI patients, ddPCR reported an overall 85.71% (12/14) (95% confidence interval [CI], 56.15 to 97.48%) sensitivity, 100% (7/7) (95% CI, 56.09 to 100.00%) and 71.43% (5/7) (95% CI, 30.26 to 94.89%) sensitivity in patients without empirical treatment and in empirically treated patients, respectively” should read “In BSI patients, ddPCR reported an overall 75.00% (12/16) (95% conﬁdence interval [CI], 47.41 to 91.67%) sensitivity, 100% (7/7) (95% CI, 56.09 to 100.00%) and 55.56% (5/9) (95% CI, 22.66 to 84.66%) sensitivity in patients without empirical treatment and in empirically treated patients, respectively.”

Page 1, abstract, lines 16 to 17: “including 10 bacteria and fungi” should read “including 13 bacteria and fungi.”

Page 3, Fig. 1: In the bottom box on the left, “ddPCR positive (*n* = 23)” should read “ddPCR positive (*n* = 21)” and “Both culture and ddPCR negative (*n* = 16)” should read “Both culture and ddPCR negative (*n* = 32).” In the bottom box on the right, “ddPCR positive (*n* = 25)” should read “ddPCR positive (*n* = 22)” and “Both culture and ddPCR negative (*n* = 50)” should read “Both culture and ddPCR negative (*n* = 63).” Figure 1 should appear as shown in this Author Correction.

**Corrected Fig 1:**
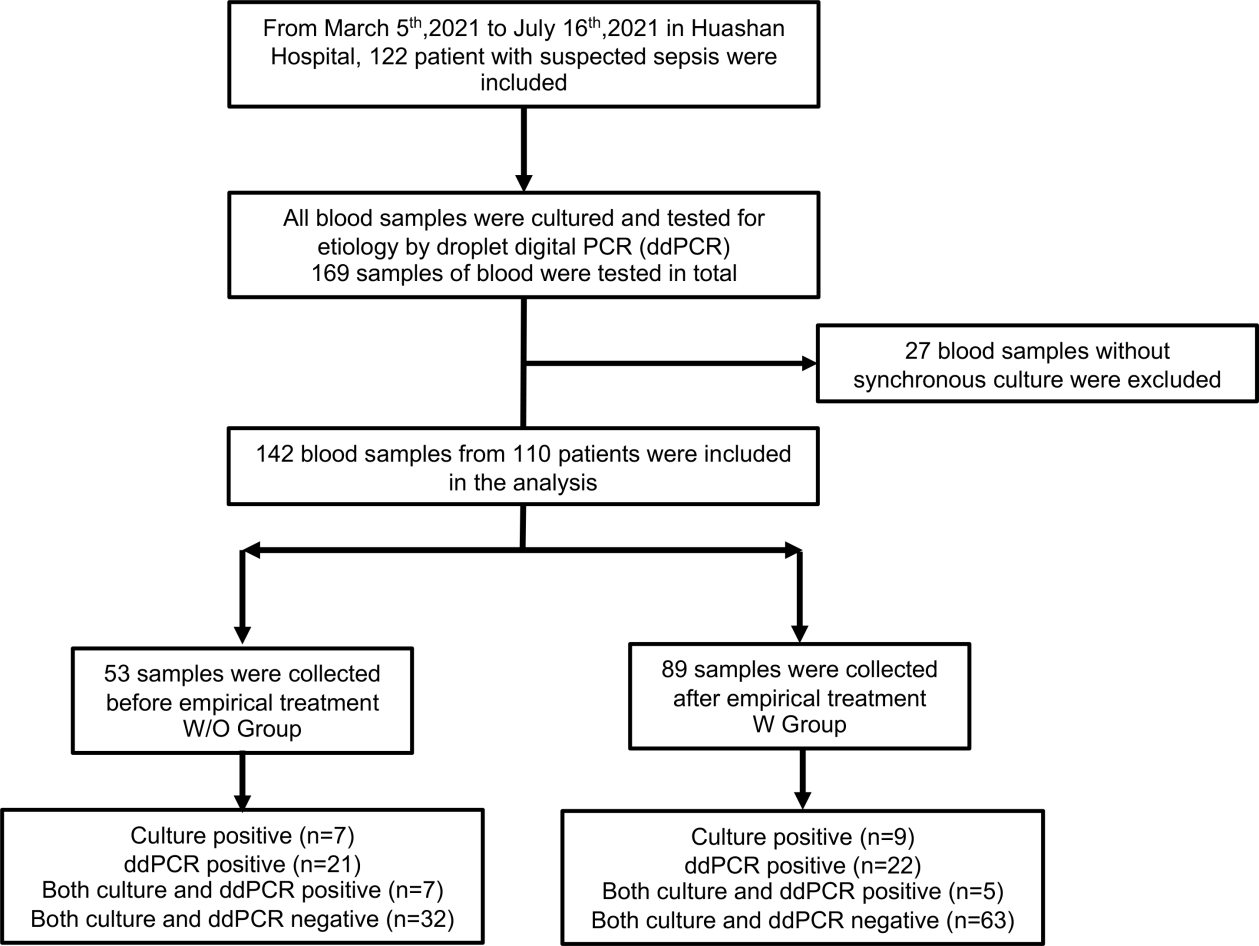


Page 5, Fig. 2A: The last row represents the numbers of cases in which *Escherichia coli* was detected by blood culture and ddPCR. The number of cases in which ddPCR test results were positive and culture results were negative (dd+/Cul−) should be 9 instead of 8, and the number of cases in which both ddPCR and culture results were positive (dd+/Cul+) should be 5 instead of 6. Panel A of Fig. 2 should appear as shown in this Author Correction.

**Corrected Fig 2A:**
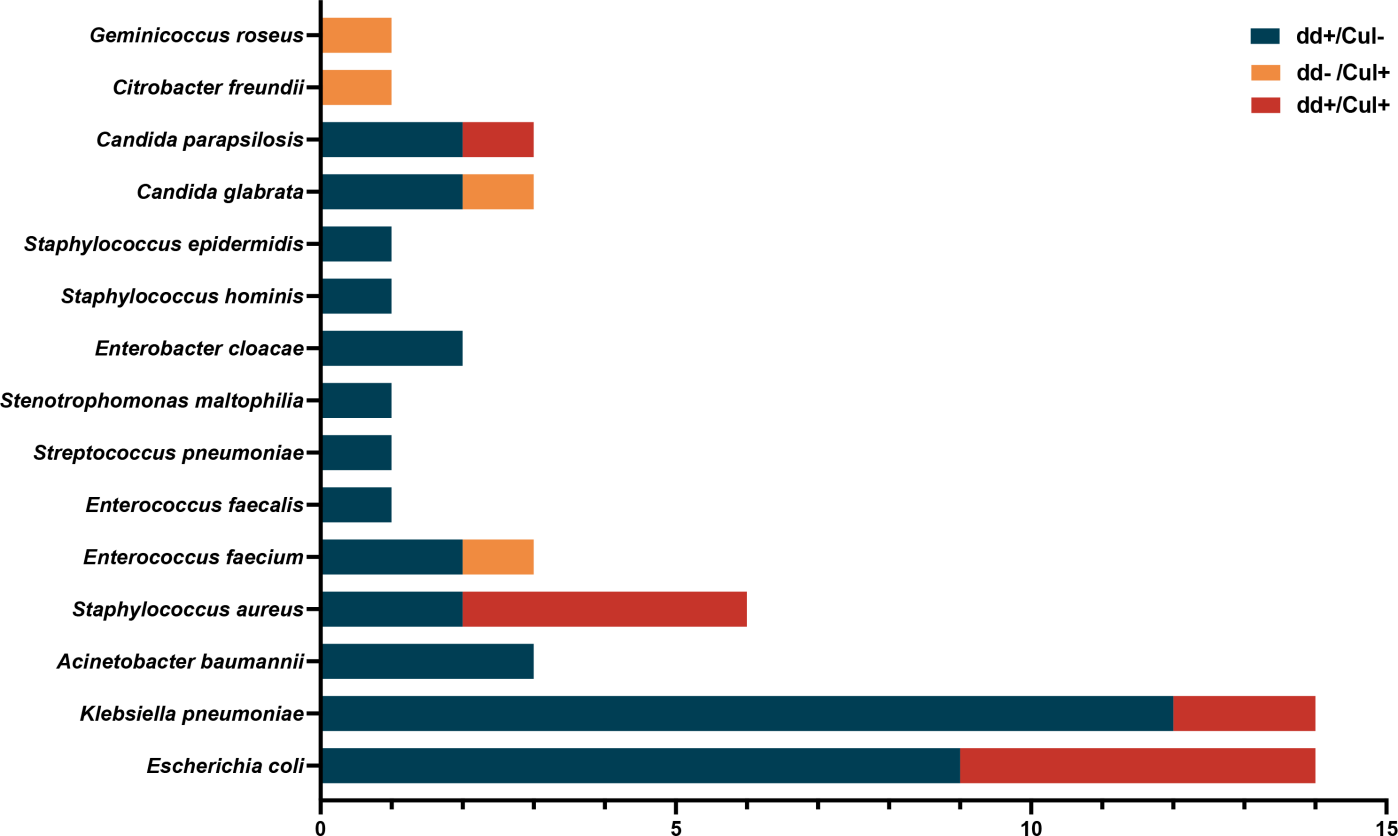


Page 5, lines 5 to 7 of the text: “and the sensitivity and specificity of the ddPCR test were both affected by the use of antibiotics (71.43% versus 100% and 78.75% versus 69.57%, respectively; *P* > 0.05)” should read “and the sensitivity and speciﬁcity of the ddPCR test were both affected by the use of antibiotics (55.56% versus 100% and 78.75% versus 69.57%, respectively; *P* > 0.05).”

Page 6, “Clinical application potential of ddPCR in antimicrobial therapy” subsection, second paragraph, lines 3 to 5: “and the regimen was kept the same. In addition, 40% (17/43) of the patients changed antimicrobial medication plans according to the ddPCR results” should read “and the regimen was kept the same for 13 of the 17 patients. In addition, for 41.9% (18/43) of the patients, antimicrobial medication plans were escalated according to the ddPCR results.”

Page 7, “Discussion” section: At the end of the first paragraph, “and 10 bacteria and fungi were detected in 31 cases with negative blood cultures” should read “and 13 bacteria and fungi were detected in 31 cases with negative blood cultures.”

Page 7, “Discussion” section: At the beginning of the third paragraph, “In this study, compared with blood culture, ddPCR had a relatively high sensitivity of 85.71% (95% CI, 56.15 to 97.48%), a specificity of 71.43% (95% CI, 30.26 to 94.89%), …” should read “In this study, compared with blood culture, ddPCR had a relatively high sensitivity of 75.00% (95% CI, 47.41 to 91.67%), a speciﬁcity of 75.40% (95% CI, 66.78 to 82.44%), …”

Page 10, Fig. 7A: In the top middle box (with the text “Initial antibiotic regimen adjusted accordingly”), “83.7% (*n* = 36)” should read “51.2% (*n* = 22).” In the bottom middle box (with the text “Initial antibiotic regimen remained the same”), “16.3% (*n* = 7)” should read “48.8% (*n* = 21).” In the top right box, “Antibiotic regimen modified by ddPCR (*n* = 18)” should read “Antibiotic regimen started/altered by ddPCR (*n* = 19).” In the second box in the right column, “Empirical antibiotic therapy (*n* = 18)” should read “De-escalated (*n* = 3).” In the third box in the right column (with the text “Keep the original treatment plan”), “(*n* = 6)” should read “(*n* = 19).” In the bottom box on the right, “Discharged without ddPCR report (*n* = 1)” should read “Discharged without ddPCR results (*n* = 2).”

**Corrected Fig 7:**
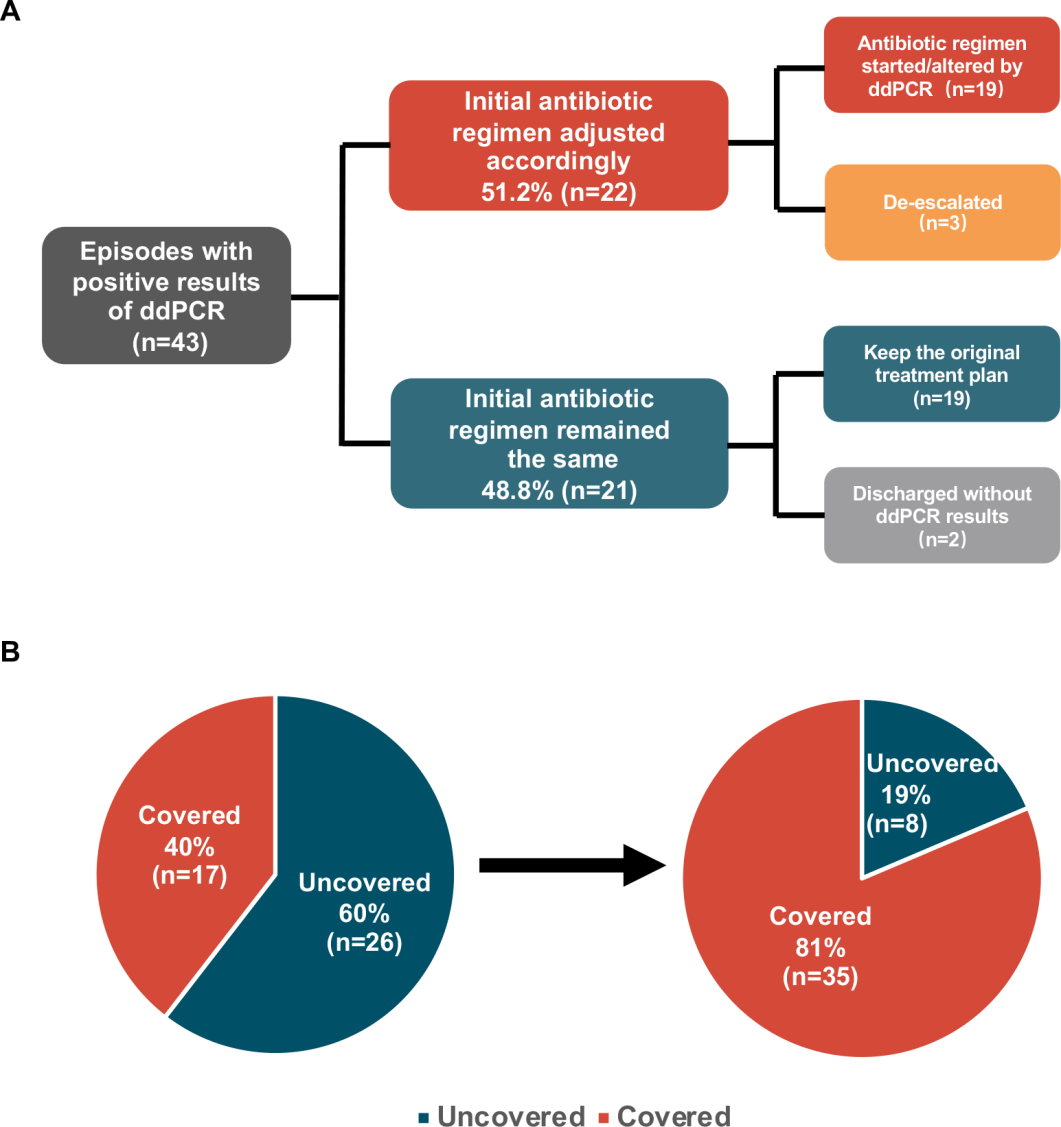


Page 10, Fig. 7B: In red area of the left pie chart, “Covered 42% (*n* = 18)” should read "Covered 40% (*n* = 17)." In the green area of the left pie chart, “Uncovered 58% (*n* = 25)” should read “Uncovered 60% (*n* = 26).” In the red area of the right pie chart, “Covered 84% (*n* = 36)” should read “Covered 81% (*n* = 35).” In the green area of the right pie chart, “Uncovered 16% (*n* = 7)” should read “Uncovered 19% (*n* = 8).”

We apologize for these errors, and the corrected data in both the abstract and discussion are now in line with the results. We state that these corrections do not change the scientific conclusions of the article in any way.

