# Supplementary material for: Clinical Diagnostic Performance of Droplet Digital PCR for Suspected Bloodstream Infections
Source: Microbiol Spectr. 2023 Jan 5;11(1):e01378-22. doi: 10.1128/spectrum.01378-22 (PMC9927361; doi:10.1128/spectrum.01378-22)

Supplementary Tables

Table S1. Risk factors of BSI

**Risk factors of BSI**
1. Advanced age (≥65 years)
2. Immunosuppression. Immunosuppressant medications including corticoids usage in the past month and comorbidities that depress host-defense (eg, neoplasms especially cancer, renal failure, hepatic failure, AIDS, asplenism). Also, diabetes and obesity were taken into consideration since they may alter the immune system, resulting in an elevated risk for developing BSIs.
3. Previously diagnosed immunodeficiency or family history.
4. Intensive care unit admission. Approximately 50 percent of ICU patients have a nosocomial infection.
5. Invasive medical procedures such as mechanical ventilation and intravascular catheter placement.

BSI, bloodstream infection. AIDS, acquired immunodeficiency syndrome. ICU, intensive care unit.

Table S2. Pathogens and AMR Genes Detection panels of ddPCR

Assay panel	Target pathogens
PilotBac-1	*A. baumannii, E. coli, K. pneumonia, P. aeruginosa,*
PilotBac-2	*E. faecalis, E. faecium, S. aureus, S. pneumoniae*
PilotBac-3	*S. capitis, S. haemolyticus, S. hominis, S. epidermidis*
PilotBac-4	*E. cloacae, P. mirabilis, S. marcescens, S. maltophilia*
PilotFungi	*C. albicans, C. glabrata, C. parapsilosis, C. tropicalis*
PilotKNA	*blaKPC, blaNDM, mecA*

AMR, antimicrobial resistance. ddPCR, digital droplet PCR.

Table S3. Concordant positive blood culture and ddPCR results and therapy adjustment when the ddPCR results became available

Microorganism detected by BC and ddPCR	Therapeutical relevance	Empirical therapy	*Feasibility of antibiotic adjustment and notes
Escherichia coli	Intervene	/	Modification necessary. Imipenem/cilastatin was added.
Escherichia coli	Intervene	/	Modification necessary. Imipenem/cilastatin was added.
Staphylococcus aureus/mecA	Intervene	Benzylpenicillin Sodium	Modification necessary. Vancomycin and Daptomycin were added.
Klebsiella pneumoniae	Intervene	/	Modification necessary. Anti-pseudomonal penicillin/tazobactam was added.
Klebsiella pneumoniae/blaKPC	Intervene	/	Modification necessary. Carbapenem and Ceftazidime/avibactam were added.
Escherichia coli	Continue	/	No change. The test was ordered on the 3rd day after a 7-day Carbapenem therapy and the result was weakly positive.
Escherichia coli	Continue	Meropenem and Fosfomycin	Empirical therapy was adequate.
Escherichia coli	Continue	Cefoperazone/sulbactam	Empirical therapy was adequate.
Staphylococcus aureus	Continue	Vancomycin and Ceftriaxone	Empirical therapy was adequate.
Candida parapsilosis	Continue	/ (Polymyxini B and Cefoperazone/sulbactam according to previous sputum culture results)	No change. The ddPCR result was considered implausible at the moment.
Staphylococcus aureus	Not relevant	/	The patient was discharged before the result became available.
Staphylococcus aureus	Not relevant	Cefazolin and Levofloxacin	The patient was discharged on the same day when the result became available.

*Feasibility of antibiotic adjustment: an empirical therapeutic regimen was deemed adequate when providing cover for the causal microorganism.

BC, blood culture. ddPCR, droplet digital PCR.

Supplementary Figure 1. Laboratory Flow. The work process of ddPCR test in Huashan Hospital.

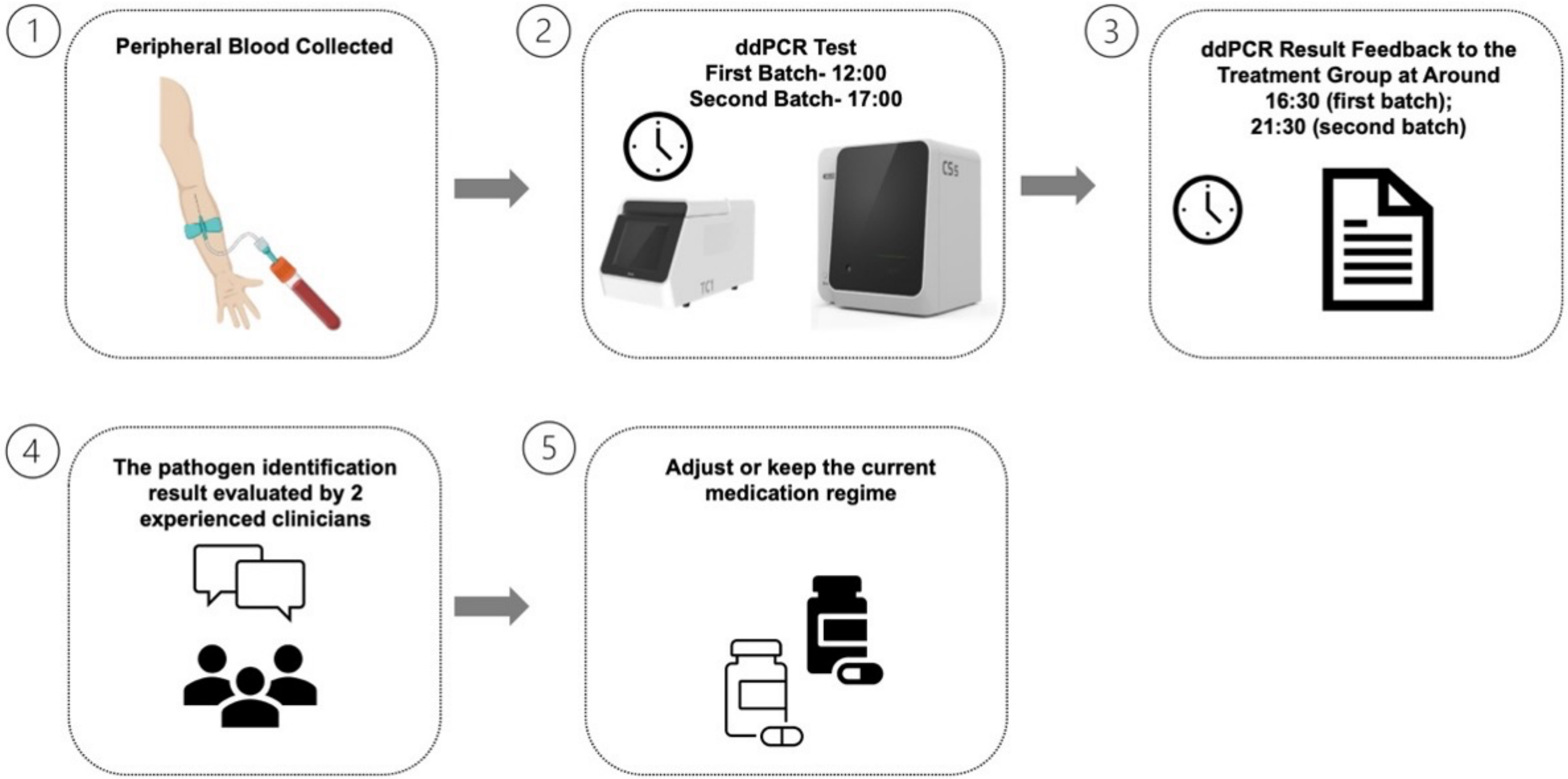

Supplement: Supplemental file 1 — Supplemental material. Download spectrum.01378-22-s0001.pdf, PDF file, 0.5 MB [file spectrum.01378-22-s0001.pdf]
